# Appropriateness of Prescription and Safety of Wearable Cardioverter Defibrillators: A Single-center Experience

**DOI:** 10.7759/cureus.4854

**Published:** 2019-06-07

**Authors:** Murtaza Sundhu, Sajjad Gul, Mubbasher A Syed, Omer Afzal, Bhavan Shah, Lon Castle

**Affiliations:** 1 Internal Medicine, Order of St. Francis - St. Francis Medical Center, Peoria, USA; 2 Cardiology, University of Toledo Medical Center, Toledo, USA; 3 Internal Medicine, University of Florida, Gainesville, USA; 4 Internal Medicine, Cleveland Clinic - Fairview Hospital, Cleveland, USA; 5 Cardiology, Cleveland Clinic - Fairview Hospital, Cleveland, USA

**Keywords:** lifevest, wearable cardioverter defibrillator, ischemic cardiomyopathy, non-ischemic cardiomyopathy, implantable cardioverter defibrillator

## Abstract

Introduction

Wearable cardioverter defibrillators (WCD) are recommended for patients with a high risk of sudden cardiac death (SCD) secondary to arrhythmia that have not qualified for placement of an implantable cardiac defibrillator (ICD). This study provides insights into a single-center experience with WCD in terms of its usage and safety.

Materials and methods

We studied all patients that were prescribed a WCD in the Fairview Hospital in Cleveland Clinic Health System, from January 2014 to June 2016. Institutional Review Board of the Cleveland Clinic approved the study. A retrospective chart review was performed to collect data regarding demographics and baseline comorbidities including age, gender, history of hypertension, diabetes, coronary artery disease, and chronic kidney disease. The patients that were lost to follow up in our electronic medical record (EMR) were excluded. Ejection fraction (EF) at the time of diagnosis and follow-up was recorded. The primary outcome was ICD placement at follow up focusing on appropriate use while the secondary outcome was delivery of shock (appropriate or inappropriate) focusing on efficacy and safety of the device. Patients were stratified based on ICD placement. Statistical Package for the Social Sciences (SPSS), version 23 (IBM Corp., NY, USA) was used for the statistical analysis.

Results

We identified 73 patients with WCD placement. After the exclusion of 23/73 (31.5%) patients due to loss of follow-up, 50 patients were included in the study (n=50). Clinical characteristics showed 66% patients were males, 76% had hypertension, 40% had diabetes, 34% had chronic kidney disease, 56% patient had a New York Heart Association functional status of >II and 34% were on anti-arrhythmic medication. Indication for WCD use was ischemic cardiomyopathy in 23/50 (46%) patients and non-ischemic cardiomyopathy in 27/50 (54%) patients. No ICD was placed in 39/50 (78%) patients and ICD was placed in 11/50 (22%) patients at end time of follow up. Mean age was 59.9 years (95% confidence interval (CI), 55.9 - 63.9 years) in the group with no ICD placement and 63.5 years (95% CI, 56.5 - 70.6 years) in the group with ICD placement. Mean EF in the group with no ICD placement at the time of diagnosis was 25.8% (95% CI, 23.8% - 27.9%) which improved by 18.8% to a mean EF of 44.6% (41.1% - 48.1%) at the follow-up. Mean EF in the group with ICD placement was 32.7% (95% CI, 27.6% - 37.9%) which reduced by 4.1% to mean EF of 28.6% (95% CI, 12.2% - 44.9%) which was statistically significant (p<0.0001). Patients who had no ICD placement were followed for an average of 162 days and with ICD placement for 78 days. There was no difference between ischemic or nonischemic groups in getting the ICD. There were no shocks delivered whether appropriate or inappropriate in our population.

Conclusion

Almost a quarter of the patients that were prescribed WCD in our center ended up with an implanted device which demonstrates appropriate use. Equally important was the observed safety of WCDs as a treatment modality with no inappropriate shocks recorded in the followed cohort.

## Introduction

Sudden cardiac death (SCD) is defined as a sudden and unexpected cessation of cardiac activity leading to compromised blood flow to the brain and other vital organs. The event is called aborted SCD (also referred to as sudden cardiac arrest) if the abnormal rhythm is reverted back to normal rhythm (spontaneously or by intervention such as defibrillation) [1]. In patients with structural heart disease, ventricular fibrillation is the most common cause of sudden cardiac arrest [1-2]. External defibrillation was found effective in terminating ventricular fibrillation and hence, implantable cardiac defibrillator (ICD) was developed and approved by the Food and Drug Authority in 1985 for secondary prevention only (which means for patients that had survived a cardiac arrest) in the vulnerable population. These devices further cemented the demonstrated reduction in mortality by converting shockable rhythms automatically in ischemic cardiomyopathy patient [3-4] and in non-ischemic cardiomyopathy patients [5]. However, a significant number of patients have an increased risk but do not qualify for implantation of ICD because of multiple reasons such as, but not limited to, unestablished chronicity of cardiomyopathy or active infection. ICDs are expensive devices that are invasive and difficult to reverse. Many arrhythmias occur early during the course of cardiomyopathy. Patients remain unprotected during this early phase as guidelines mandate a waiting period to ensure that the deterioration in cardiac function is irreversible. Wearable cardioverter defibrillator (WCD) is a device which can detect and treat the ventricular tachycardia (VT) and ventricular fibrillation. WCD has been presented as the solution to bridge this vulnerable population to the implantable device during the waiting period. WCD is a vest which is worn and requires custom fitting to avoid electrical noise. It detects and defibrillates/cardioverts the abnormal rhythms through electrical electrodes.

The purpose of this study was to analyze our experience at Fairview Hospital, a community hospital in the Cleveland Clinic Health System, in terms of appropriateness of prescription of this wearable device and outcomes reflecting the safety and efficacy of this device. New technologies often have to overcome many hurdles in the phase of early adoption. One of these is an inappropriate prescription as the prescriber’s grapple with the appropriate indications for the device. Another common issue is the safety of new devices that are also the focus of post-marketing analyses. We sought to review our records for a two-year period to make an assessment regarding these variables with WCD usage at our center.

## Materials and methods

Study design

This was a retrospective observational study which was conducted from January 2014 to June 2016. The study was conducted on patients admitted to Fairview Hospital in Cleveland. The study was approved by the Institutional Review Board of the Cleveland Clinic and the individual consent was waived as it was a retrospective study.

Seventy-three patients that were prescribed and fitted with the WCD (LifeVest®, ZOLL, Pittsburgh, Pennsylvania) during this period were identified. The inclusion criteria comprised of patients with age more than 18 and with a documented diagnosis indicating the prescription of a WCD based on documented ejection fraction (EF). The exclusion criteria included people who had an ICD, congenital heart disease, and patients who were lost to follow-up. Patient's records were reviewed for any delivered shocks and then validated with Zoll© (manufacturer of LifeVest®). After excluding 23 patients due to lack of adequate follow up, the final sample size of 50 (n=50) was analyzed for safety and efficacy outcomes.

Data collection

We collected data in Microsoft Excel and data safety was ensured by the usage of IronKey© for data sharing. Data was collected for age, gender, race, diagnosis, diabetes, hypertension, chronic kidney disease, history of coronary artery disease, history of congestive heart failure, use of antiarrhythmic drugs, last cardiac catheterization, findings on cardiac catheterization, EF, source of EF, and fit date for the WCD. Follow-up was assessed with the follow-up date, follow-up EF, and difference in the EF. The primary outcome was eventual ICD placement and the secondary outcome was delivery of shock, including both appropriate and inappropriate.

Statistical analysis

Data was analyzed using Statistical Package for the Social Sciences (SPSS), version 23 (IBM Corp., NY, USA). Fisher’s exact method was used for the categorical variables and analysis of variance (ANOVA) was used for the continuous variables.

## Results

The total number of patients included in the study were 50 (n=50). The indication for prescription of WCD was ischemic cardiomyopathy in 46% (23/50) of patients and non-ischemic cardiomyopathy in 54% (27/50) of patients. The descriptive statistics showed that the mean age of the population was 60.7 years, 66% (33/50) of patients were male, 64% (32/50) were Caucasian, 40% (20/50) had diabetes, 76% (38/50) had hypertension, 34% (28/50) had chronic kidney disease, 56% (28/50) had coronary artery disease, 32% (16/50) had New York Heart Association category III or IV. The median follow-up time was three months (103.5 days) and the mean follow-up was 150 days (Table 1).

**Table 1:** Baseline characteristics and demographics of the sample

Variable		n	Percent %
Gender	Female	17	34
	Male	33	66
Indications for Wearable Cardioverter Defibrillator	Ischemic	23	46
	Non-Ischemic	27	54
Implanted Cardioverter Defibrillator (ICD) placed or Not	No ICD	39	78
	ICD placed	11	22
History of Hypertension	No	12	24
	Yes	38	76
History of Diabetes Mellitus	No	30	60
	Yes	20	40
History of Chronic Kidney Disease	Absent	33	66
	Present	17	34
Patients on Anti-arrhythmic Medication	No	33	66
	Yes	17	34

The patients were divided into two groups i.e., with ICD placement or without ICD placement, based on whether implantable cardioverter defibrillator was inserted or not. ICD was placed in 22% (11/50) patient and no ICD was placed in 78% (39/50) patients. Mean left ventricular ejection fraction (LVEF) in the group with ICD placement was 32.7% (95% confidence interval (CI); 27.6% - 37.9%) which decreased by 4.5% to reach a mean LVEF of 28.6% (95% CI; 12.2% - 44.9%) compared to the group without ICD placement which had a mean LVEF of 25.9% (95% CI; 23.8% - 27.9%) and it increased by 17.3% to reach a mean LVEF of 44.6% (95% CI; 41.1% - 48.2%) with a statistical significance in ANOVA (Table 2). Patients in the ICD group were followed for a mean of 78 days (range; 61 - 96) compared to No ICD placed group which were followed for a mean of 162 days (range; 104 - 220).

**Table 2:** Comparison outcomes between the two groups based on eventual implantation of a permanent device ICD: implantable cardiac defibrillator.

		Mean	95% Confidence Interval for Mean		P Value
			Lower Bound	Upper Bound	
Age	No ICD	59.949	55.963	63.935	0.382
	ICD placed	63.545	56.465	70.626	
	Total	60.740	57.351	64.129	
Ejection Fraction on Fit Date	No ICD	25.868	23.798	27.939	0.005
	ICD placed	32.778	27.639	37.917	
	Total	27.191	25.173	29.210	
Ejection Fraction at last follow up	No ICD	44.629	41.083	48.174	0.003
	ICD placed	28.600	12.207	44.993	
	Total	42.625	38.847	46.403	
Difference between Ejection fraction	No ICD	17.259	13.172	21.346	<0.000
	ICD placed	-4.500	-13.148	4.148	
	Total	13.303	8.680	17.926	
Days between follow up	No ICD	162.20	104.01	220.39	
	ICD placed	78.67	61.20	96.13	
	Total	149.98	99.75	200.20	

## Discussion

The current recommendations for WCD come from the American Heart Association, American College of Cardiology and Heart Rhythm Society guidelines for the management of patients with ventricular arrhythmias and prevention of SCD [6]. Currently WCD is placed within 40 days of a new myocardial infarction with low LVEF, reduced EF (less than 35%) in a patient within 90 days post coronary artery bypass grafting (CABG), potentially reversible nonischemic cardiomyopathy with severely reduced LVEF less than 35%, and severe heart failure patients waiting for heart transplant [6].

Our study investigated 50 patients with both ischemic and non-ischemic cardiomyopathy with EF lower than 35%. The patients were on guideline-directed medical therapy (GDMT) while wearing WCD and only 22% patients ended up needing an ICD implantation per ACC/AHA guidelines while 78% had significant improvement in EF and did not require placement of an ICD. None of the patients received a shock from the WCD and all patients survived. The ICD placement group had reduction in EF which was due to the progression of the underlying disease process despite being on GDMT. The number needed to treat could not be calculated as no treatments were dispensed and this was perhaps due to the limitation imposed by the small sample size. The patients in the no ICD placement period were followed for a longer duration until there was a documented increase in EF which did not warrant the placement of ICD.

Vest Prevention of Early Sudden Death Trial (VEST trial) reported that the rate of arrhythmic deaths did not differ in patients wearing a WCD while on GDMT compared to those who were only taking GDMT and not wearing WCD. This trial targeted the same demographic as our analysis, and investigated patients soon after an acute myocardial infarction with reduced EF of 35% or lower [7]. However, the trial was considered underpowered to determine the beneficial effect of WCD. A recent meta-analysis by Masri et al. [8], that included 27 observational studies and one randomized controlled trial (RCT) which was the VEST trial mentioned above, demonstrated that mortality in patients wearing WCD was rare at 0.7 per 100 patients in first three months. The study concluded that appropriately treated patients with WCD were higher in observational studies than the VEST trial owing to the significant heterogeneity in the methodology of the included studies.

VEST trial demonstrated the inconvenience of using WCD as a major limitation for the device. This was manifested as a lower than expected adherence to WCD use in a closely followed sample [7]. In our study, all patients reported compliance with WCD but follow up was admittedly less focused on investigating device adherence than in VEST trial.

Moreover, inappropriate shocks are a major concern for ICDs as they occur in about 40% of patients with ICD [9-10] and there is an increased risk of death in patients who received shock appropriately (for VT) or inappropriately [11-12]. In the cohort we studied, there were no inappropriate shocks reported during the course of follow up and this endorses the safety of this device. A study detailing three-year experience with WCDs, published from France, did not show any appropriate shocks and one inappropriate shock which is similar to the results we saw in a much smaller sample [13]. There was another study published out of Germany that analyzed six years of data and reported one appropriate and two inappropriate shocks [14].

The current review of the literature and our findings suggest that more studies are needed to effectively report the efficacy and safety of WCD for primary prevention of life-threatening arrhythmias.

## Conclusions

Almost a quarter of the patients that were prescribed WCD in our center ended up with an implanted device which demonstrates appropriate use. Equally important was the observed safety of WCDs as a treatment modality with no inappropriate shocks recorded in the followed cohort.
